# Investigating the relationship between district-level socioeconomic status and individual obesity in Taiwanese adolescents: A large-scale cross-sectional analysis

**DOI:** 10.1038/s41598-019-39167-5

**Published:** 2019-02-27

**Authors:** Ying-Lien Ni, Jen-Ho Chang, Lung Hung Chen

**Affiliations:** 1 Department of Physical Education, Health & Recreation, National Chiayi University, Chiayi, Taiwan; 2 Institute of Ethnology, Academia Sinica, Taipei, Taiwan; 3 Department of Psychology, National Taiwan University, Taipei, Taiwan; 4 Department of Recreation and Leisure Industry Management, National Taiwan Sport University, Taoyuan, Taiwan

## Abstract

The current study aimed to assess the prevalence of obesity and to explore the relationship between socioeconomic status and obesity among adolescents in Taiwan, a transitioning country. Data from the Taiwan School Physical Fitness Database on 1,875,627 Taiwanese adolescents aged 10–18 years were analyzed. The average family income per household in each district was collected from the national statistical institutional database. Descriptive statistics, Chi-square tests, Pearson correlation analysis, and mixed model analyses were used. The overall prevalence of combined overweight and obesity was 28.1%. The prevalence of overweight/obesity significantly differed according to gender and age. Furthermore, the average family income per household was negatively associated with the district-level prevalence of obesity. Additionally, when controlling for physical fitness, the average family income per household remained negatively associated with adolescent obesity. In addition, multilevel analysis was also applied to explore the relationship between district-level socioeconomic status and individual-level obesity to prevent the nested data structure from affecting the results. The results revealed that the average family income per household negatively correlated with individual obesity. These findings provide insight for public health officials into preventing and managing adolescent obesity.

## Introduction

Obesity is a serious public health challenge that is associated with substantial costs in both developing and developed countries^1–4^. Identifying the risk factors of obesity may thus represent an important aspect of public health. Previous research has indicated that socioeconomic status is closely related to the risk of obesity in adolescent populations^5^. However, socioeconomic status and obesity have shown differing associations depending on the level of country development. Specifically, a negative relationship has been found in most developed countries, whereas a positive relationship has been observed in developing countries^6,7^. Although the associations between socioeconomic status and adolescent obesity have been well established in developing and developed countries, little attention has been paid to adolescents in newly developed countries such as Taiwan. Accordingly, to address this gap, a social ecological perspective may be suitable for exploring the relationship between socioeconomic status and the current obesity epidemic. We conducted a large-scale analysis of a nationally representative sample of young adolescents in the most current Taiwan study to date, with the aims of investigating the relationship between socioeconomic status and adolescent obesity and understanding how socioeconomic status promotes weight gain in a country in economic transition.

## Socioeconomic status and obesity in developed and developing countries

Empirical evidence has shown a negative relationship between socioeconomic status and obesity among adolescents in developed countries, such as the United States^8^, England^9^, and Canada^10^. In other words, adolescents in developed countries who are raised in a low socioeconomic status setting are more likely to be overweight or obese than those who grew up in a high socioeconomic status setting^6,11,12^. Studies have suggested several possible explanations for this negative relationship. For instance, young adolescents from high socioeconomic backgrounds tend to have abundant resources and educational advantages, and they consequently have more opportunities to choose healthy foods, cultivate healthy behaviors such as involvement in sports or physical activities, and gain scientific health knowledge that can help them maintain a normal weight^6,7^. In contrast, adolescents with a low socioeconomic status may be less likely to be able to afford healthy foods and may not receive the health benefits of maintaining a proper diet, exercise, or weight, which can lead to excessive weight gain^6,10^. As mentioned above, growing evidence has described how socioeconomic status contributes to the prevalence of obesity among adolescents in developed countries.

In contrast, in developing countries, a positive relationship between socioeconomic status and obesity among adolescents has emerged. Adolescents with a high socioeconomic status were more likely to be obese than those with a low socioeconomic status^6,8,11^. According to a recent systematic review, the evidence unanimously depicts adolescent obesity as more prevalent among high socioeconomic status populations in developing countries, such as India, Vietnam, Guatemala, and Ukraine^8,13^. Several potential explanations for this relationship have been proposed. Specifically, young adolescents with a high socioeconomic status may be more able to afford and demand surplus food and avoid physical labor, thus leading to obesity, whereas low socioeconomic status adolescents may face food shortages and increased amounts of physically demanding labor, which could help prevent obesity and could even enable a lean body mass index (BMI)^7^. In addition, cultural values in developing countries can affect obesity. For instance, excess weight can symbolize a high status and thus power and strength; therefore, people with a higher socioeconomic status may prefer a larger body size, leading to their higher BMI^7,13^. In short, socioeconomic status has shown a positive association with adolescent obesity in developing countries.

## Unclear relationship in newly developed countries

Despite growing concern regarding the relationship between socioeconomic status and adolescent obesity in developed and developing countries, little research has been conducted on this relationship in countries in economic transition. In the last few decades, Taiwan has undergone an economic transition from a developing to a developed country^14^. Although the International Monetary Fund classifies Taiwan as a developed country^15,16^, Taiwan is considered a developing country by the United Nations (as of 2014)^17^. Other international organizations have reported that Taiwan’s per capita gross domestic product (GDP) is strong and that its economy is diversified^16^. Thus, Taiwan’s economic and quality of life metrics seem to support its status as a developed country. However, compared to highly developed countries, such as the United States, Canada, the United Kingdom, and European countries including Germany and France, Taiwan is still a relatively newly developed country that is in a period of rapid economic transition and development.

In Taiwan, obesity has become increasingly prevalent among adolescents in the past two decades, and it has thus become a major public health concern^18–22^. Furthermore, the healthy, traditional diets and sedentary behavior of adolescents in Taiwan have been affected by the Western lifestyle^23,24^. However, empirical evidence on the relationship between socioeconomic status and adolescent obesity is lacking in countries in economic transition, such as Taiwan. According to the International Monetary Fund, Taiwan’s income status increased progressively from 1970–2010^16^. Consistent with the relationship between socioeconomic status and obesity in developed countries, high socioeconomic status adolescents appear to be more inclined to recognize the value of health and thus to access healthy foods and education resources than low-socioeconomic status adolescents; consequently, a higher socioeconomic status may contribute to a lower risk of obesity in Taiwanese adolescents. Thus, we hypothesized that in the current transitional economy and society in Taiwan, socioeconomic status would be negatively associated with adolescent obesity.

Because researchers mentioned that family income has been more widely used as an indicator of socioeconomic status^25,26^, average family income per household was adopted to identify socioeconomic status in the present study. Moreover, prior studies have indicated that the propensities for overweight and obesity can vary by the amount of physical activity performed and that physical fitness can modify the effects of socioeconomic status^20,27,28^. Accordingly, we conducted partial correlations to exclude the confounding effects of physical fitness (flexibility, muscular endurance, anaerobic power, and aerobic fitness) and investigated the relationship between socioeconomic status and obesity.

As mentioned above, the association between socioeconomic status and obesity has been extensively investigated in developing and developed countries. For example, some studies have focused on the individual level to explore this relationship^6,9,12^, while others explored this relationship at the district level or national level^8^. However, individuals are nested within towns, states, or countries. Thus, studies are concerned only with the correlation between socioeconomic status and obesity at the individual level may not fully understand how socioeconomic status influences obesity because of how the nested data structure may affect the results. To circumvent this limitation in the previous research, recent studies have begun to adopt a multilevel perspective to explain the relationship between socioeconomic status and obesity^29,30^. As such, in the present study, multilevel analysis is also applied to explore the relationship between district level socioeconomic status and individual level obesity.

Overall, the first aim of this study was to examine the most recent data on the current national prevalence of obesity among Taiwanese adolescents. In addition, the second aim was to clarify the underlying relationships between socioeconomic status and adolescent obesity in Taiwan. We sought to determine these relationships at both the district level and the individual level. The results of this study will provide a better understanding of the prevalence of adolescent obesity and will expand our knowledge of how socioeconomic status is associated with obesity among adolescents in Taiwan, a country undergoing rapid economic and social transition. Furthermore, these findings may provide important implications to policy makers and intervention designers for the development of effective strategies to improve the prevention and management of overweight and obesity among adolescents.

## Methods

A cross-sectional study was conducted to determine the prevalence of obesity and the relationship between socioeconomic status and obesity prevalence among Taiwanese adolescents aged 10 to 18 years in the 2014 school year. Ethical approval for this study was obtained from the Sports Administration, Ministry of Education, Taiwan (Letter Number: 1050004628). Accordingly, all methods were performed in accordance with the institution’s relevant guidelines and regulations. In addition, participant consent was not necessary because this study involved the use of a previously published deidentified database according to Sports Administration, Ministry of Education. Because a deidentified database is used in this study, the informed consent requirement was waived in compliance with the Department of Health, Executive Yuan standards (No.1010265083).

### Data sources and study sample

**Figure 1:**
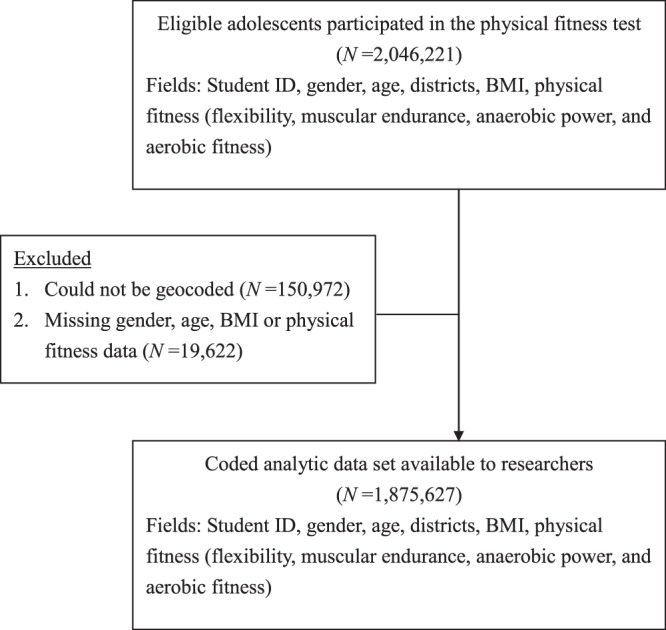
Data acquisition flow diagram.

We merged national physical fitness data and sociodemographic data from two nationwide administrative databases. The Taiwan School Physical Fitness Database was developed by the Ministry of Education in 1999 and includes data such as measured height, weight, and physical fitness indices of school adolescents aged 7 to 18 years. This database provides the unique opportunity to study the prevalence of overweight and obesity and to monitor the physical fitness levels of school adolescents. Details on the Taiwan School Physical Fitness Database have been described previously^20,31^. Briefly, the Taiwan School Physical Fitness Database is a school-based health-related physical fitness surveillance system that includes data provided each year by school teachers after students have undergone physical fitness tests, and these data are then managed by the Ministry of Education. The Taiwan School Physical Fitness Database contains information on each adolescent’s gender, age, height, weight, BMI, physical fitness indicators, grade level, and school location. A total of 2,046,221 adolescents participated in the physical fitness tests from September 2014 to June 2015. We included only adolescents who had complete data for all key measures and covariates; thus, the final dataset consisted of 1,875,627 adolescents **(**892,946 girls, 47.6%**)** whose height, weight, and indices of physical fitness (flexibility, muscular endurance, anaerobic power, and aerobic fitness) were objectively measured and included for analysis in the current study. Figure 1 presents the data acquisition process. In addition, the variable indicating socioeconomic status was obtained from the Directorate General of Budget, Accounting and Statistics of Executive Yuan of Taiwan, which is a national statistical institute that is responsible for collecting and publishing statistics related to the population and economy at the national, regional and local levels each year. We collected average family income per household data for 2014 in 19 districts in Taiwan (6 cities and 13 counties) from the government survey on family income and spending^32^.

### Definition of variables

The study variables included demographic characteristics (gender and age), BMI, obesity rates, physical fitness (flexibility, muscular endurance, anaerobic power, and aerobic fitness), and average family income per household.

BMI is a measure of body fat that is based on height and weight and is calculated as weight/height^2^ (kg/m^2^). The BMI criteria for obesity among Taiwanese children and adolescents, based on the World Health Organization standards and ethnic differences, were used to classify the participants as underweight (BMI ≤ 4th percentile), normal weight (5th percentile ≤ BMI ≤ 84th percentile), overweight (85th percentile ≤ BMI ≤ 94th percentile) or obese (BMI ≥ 95th percentile) according to age and gender^33^. The prevalence of obesity was the percentage of overweight and obesity in a given district.

The components of physical fitness according to the Ministry of Education guidelines included flexibility (sit-and-reach), muscular endurance (sit-ups), anaerobic power (standing long jump), and aerobic fitness (run/walk test)^34^. A modified sit-and-reach assessment was used to evaluate lower back and hamstring flexibility. The adolescents removed their shoes and sat on the floor with their legs fully extended at right angles with the zero point of a 25-cm scale. They were instructed to place one hand on top of the other and to then slowly reach forward along the top of the ruler as far as possible with their palms facing downward. Each student performed the test twice, and the best stretching distance was recorded in centimeters (cm). Muscular endurance was assessed by a timed bent-leg sit-up test. Adolescents lay on a mat, crossed their arms across their chest while curling up to a sitting position until their elbows touched their knees, and then returned to the floor. Each student performed as many sit-ups as possible within 60 seconds. The maximum number of correct sit-ups in 60 seconds was recorded. The standing long jump test, which assessed anaerobic power, was performed as a two-footed take-off and landing. Each adolescent was instructed to stand behind a starting line with their feet shoulder-width apart and to jump forward as far as possible. The best score of two attempts was recorded in centimeters (cm). Aerobic fitness was evaluated by a run/walk test. All girls and boys aged 10–12 years participated in an 800-m run/walk test, and a 1600-m run/walk test was conducted for boys aged 13 to 18 years. Adolescents were encouraged to do their best to complete the run/walk test as fast as possible. Time to complete the test was measured and recorded in seconds. Those data were the individual level physical fitness. In addition, we calculated the average individual physical fitness in each district to represent district-level physical fitness.

Furthermore, according to the Directorate General of Budget, Accounting, and Statistics of Executive Yuan of Taiwan^32^, average family income per household (unit: NT) was defined as the annual average of total receipts, comprising employee compensation, entrepreneurial income, property income, imputed rent income, current transfer receipts, and miscellaneous receipts. The average family income per household in each district was calculated by dividing the total family income in each city or county by the total households in each city or county. Based on the definition of average family income per household in form the national statistical institute, we collected each district’s average family income per household to identify socioeconomic status at the districts levels.

### Statistical analysis

First, descriptive statistics for gender, age, weight, height, BMI, physical fitness indicators, and average family income per household were calculated. Differences in the prevalence of overweight and obesity were assessed by age group and gender using Chi-square tests. Second, to explore the relationship between socioeconomic status and obesity, we aggregated the individual variables into the city and county levels. Then, Pearson correlation coefficients were computed to examine the associations between average family income per household and obesity rates at the district level. We also applied partial correlation coefficients to exclude the confounding effects of physical fitness to investigate the relationship between socioeconomic status and obesity at the district level. Furthermore, to circumvent the weakness of single-level analysis in prior studies, we applied multilevel analysis to explore the relationship between district-level socioeconomic status and individual-level obesity. As such, mixed-model analysis was used to explore the relationship between district-level average family income per household and BMI at the individual level. Data were analyzed using PASW statistical package version 18.

### Materials and Correspondence

Data are available from the Institutional Ministry of Education Data Access for researchers who meet the criteria for access to confidential data. LHC is the corresponding author who will respond to correspondence and material requests.

## Results

The descriptive statistics obtained in the current study are presented in Table 1.

**Table 1:** Descriptive statistics of the study (N = 1,875,627).

Variable	Mean	*SD*
Age (years)	13.96	2.43
Weight (kg)	51.81	14.74
Height (cm)	157.42	11.75
BMI (kg/m^2^)	20.60	4.18
**Physical fitness**		
Sit-and-reach (cm)	29.24	9.95
Sit-up (times)	33.92	10.73
Standing long jump (cm)	166.73	36.09
Run/walk (sec)	376.75	146.78
**Socioeconomic status (SES)**		
Average family income per household (New Taiwan dollar)	1,130,072	259,695.15

### Body mass index and the prevalence of overweight and obesity

**Figure 2:**
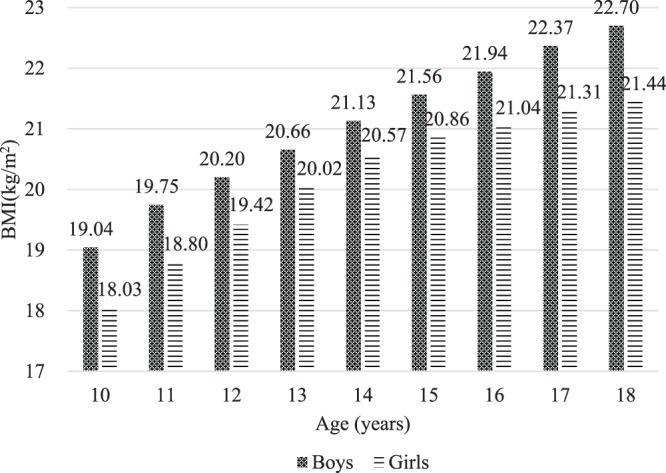
Average body mass index (BMI) of adolescents aged 10–18 years.

The mean BMI was 21.04 kg/m^2^ (*SD* = 4.45) for boys and 20.13 kg/m^2^ (*SD* = 3.80) for girls. Overall, the average BMI was higher in boys than in girls at each age. The average BMI of adolescents aged 10–18 years are presented graphically in Fig. 2.

Furthermore, Table 2 summarizes the distribution of obesity, overweight, normal weight, and underweight by gender and age. The overall prevalence of combined overweight and obesity among adolescents was 28.1%. Boys were more likely to be overweight/obese than girls (32.1% vs. 23.6%). In each age group, over a quarter of adolescents were overweight or obese (ranging from 24.4% to 31.2%). The prevalence of overweight and obesity among adolescents decreased with age, from 10 to 18 years. According to the results, the prevalence of overweight/obesity significantly differed between boys and girls (χ^2^(3) = 20496.53, *p* < 0.001) and by age (χ^2^(24) = 16178.97, *p* < 0.001).

**Table 2:** The distributions of obese status determined by BMI and stratified by age and gender (N = 1,875,627).

	Total	Obese		Overweight		Normal weight		Underweight		Overweight or obese		
		*N*	%	*N*	%	*N*	%	*N*	%	*N*	%	*p* value
Gender												<0.001
Boys	982681	182080	18.5	133105	13.5	577580	58.8	89916	9.2	315185	32.1	
Girls	892946	104584	11.7	106356	11.9	600105	67.2	81901	9.2	210940	23.6	
Age												<0.001
10	178780	28345	15.9	25972	14.5	108724	60.8	15739	8.8	54317	30.4	
11	195644	33985	17.4	27085	13.8	119577	61.1	14997	7.7	61070	31.2	
12	213711	34802	16.3	28371	13.3	132997	62.2	17541	8.2	63173	29.6	
13	222828	34583	15.5	28588	12.8	143671	64.5	15986	7.2	63171	28.3	
14	258921	38945	15.0	29857	11.5	169461	65.4	20658	8.0	68802	26.6	
15	254532	37573	14.8	30585	12.0	163926	64.4	22448	8.8	68158	26.8	
16	190440	28400	14.9	23171	12.2	119893	63.0	18976	10.0	51571	27.1	
17	206617	32219	15.6	26095	12.6	128512	62.2	19791	9.6	58314	28.2	
18	154154	17812	11.6	19737	12.8	90924	59.0	25681	16.7	37549	24.4	

### Correlations between socioeconomic status and obesity

To assess the relationship between socioeconomic status and obesity at the district level, we first aggregated individual BMIs to determine the prevalence of obesity for each city and county to examine the simple correlation at the district level. Moreover, we adopted multilevel analysis to examine the relationship between average family income per household at the district level and individual BMI through mixed model analyses.

**Figure 3:**
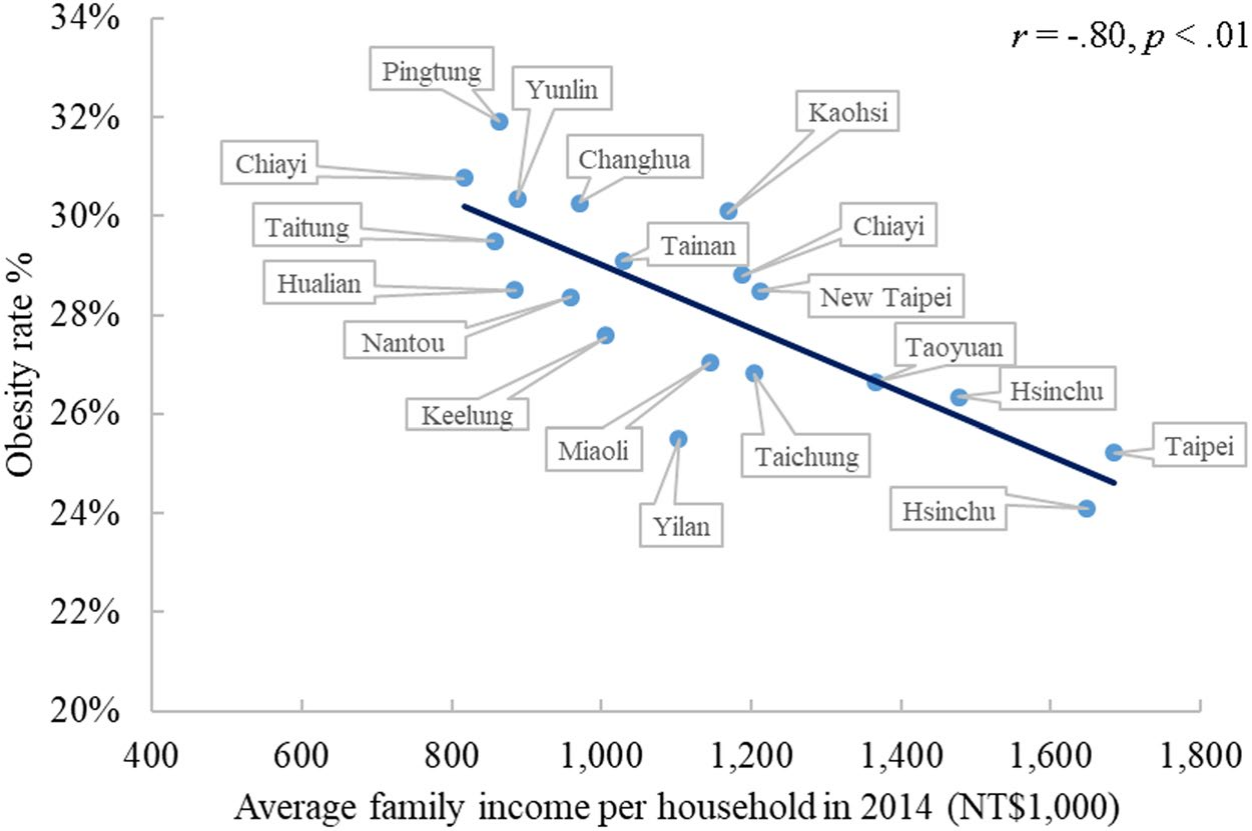
Correlation between average family income per household and prevalence of obesity at the district level.

The results revealed a strong negative association between average family income per household and the rate of obesity at the district level (*r* = −0.80, *p* < 0.01), as presented in Table 3. A more detailed understanding of this relationship is demonstrated in Fig. 3. The results also indicated that average family income per household was negatively associated with the prevalence of obesity in both boys and girls (*rs* = −0.65 to −0.87, *ps* < 0.01). Additionally, as expected, this pattern of association was observed in each age category (*rs* = −0.63 to −0.78, *ps* < 0.01). The findings suggested that young adolescents from districts with a low socioeconomic status had an increased prevalence of overweight and obesity.

**Table 3:** Correlations between socioeconomic status and obesity rates at the district level (N = 1,875,627).

Age (years)	Obesity rates	
	Not controlled for physical fitness	Controlled for physical fitness
10	−0.69***	−0.51*
11	−0.74***	−0.66***
12	−0.76***	−0.73***
13	−0.70***	−0.54**
14	−0.71***	−0.44*
15	−0.68***	−0.48*
16	−0.63***	−0.32
17	−0.67***	−0.70***
18	−0.78***	−0.52*
Total	−0.80***	−0.50*

*p < 0.10; **p < 0.05; ***p < 0.01.

As mentioned before, physical fitness may confound the effects of socioeconomic status. Accordingly, we conducted partial correlations to exclude the confounding effects of physical fitness to investigate the relationship between socioeconomic status and obesity at the district level. The results indicated that after controlling for physical fitness indicators, the average family income per household was significantly negatively associated with the prevalence of obesity (*r* = −0.50, *p* < 0.10), as indicated in Table 3. Although there was a slight decrease in the strength of the relationship between socioeconomic status and obesity in each age group, the negative correlation pattern remained. Detailed correlation coefficients are given in Table 3.

In addition, to prevent the nested data structure from affecting the correlates of socioeconomic status and obesity, mixed-model analysis was further applied to explore the multilevel relationship between district-level socioeconomic status and individual-level obesity. For this regression model, district-level socioeconomic status was used to predict individual-level obesity. As shown in Table 4, according to the fixed effect results, district-level family income per household significantly predicted individual level obesity (*b* = −7.34, *p* < 0.01); that is, a higher family income per household was associated with a lower BMI in all districts. Moreover, after controlling for physical fitness performance, the mixed-model analysis results indicated the same pattern: higher family income per household was associated with a lower BMI (*b* = −6.38, *p* < 0.01). To estimate the differences between districts in the effect of district-level family income per household on individual-level BMI, the variance of this random effect was also tested. The findings revealed only a 0.2% (0.02/(0.02 + 16.15)) variance in BMI across districts, which indicates that the negative relationship between socioeconomic status and obesity in Taiwanese adolescents in the present study does not differ across districts.

**Table 4:** Multilevel analysis in predicting individual-level BMI.

Fixed effects	Not controlled for physical fitness			Controlled for physical fitness		
BMI	Coefficient	S.E.	t/Wald Z	Coefficient	S.E.	t/Wald Z
Intercept	16.74	0.14	121.39***	17.89	0.13	140.38***
Gender	−0.90	0.01	−153.33***	−1.04	0.01	−123.86***
Age	0.43	0.001	357.04***	0.50	0.001	362.39***
Average family income per household	−7.34	1.18	−6.21***	−6.38	1.08	−5.91***
Sit-and-reach				0.05	0.001	153.06***
Sit-up				−0.02	0.001	−50.34***
Standing long jump				−0.04	0.001	−316.83***
Run/walk				0.01	2.53	331.66***
Variance component						
Level 2- district	0.02	0.01	2.85***	0.01	0.005	2.82***
Level 1- individual BMI	16.15	0.02	968.40***	13.99	0.01	968.40***
−2*log-likelihood	1.05			1.03		

*p < 0.10; **p < 0.05; ***p < 0.01.

## Discussion

Obesity is a growing problem that has been increasing in prevalence in children and adolescents worldwide^5,35^. As mentioned in the Introduction, socioeconomic status is an important factor that contributes to adolescents’ risk of obesity^5^. Several studies have examined the relationship between socioeconomic status and obesity in developing and developed countries^6,8–11,13,36^; however, few have reported on the link between socioeconomic status and obesity in a country with a transitional economy and society such Taiwan. To address this gap, our cross-sectional study used a large-scale analysis to examine the prevalence of overweight and obesity and the relationship between socioeconomic status and obesity among Taiwanese adolescents. The present study provides several contributions that can be summarized as follows. First, we found that the current prevalence of overweight and obesity among Taiwanese adolescents was higher than in the past. Second, the current study showed that in a newly developed country such as Taiwan, lower socioeconomic status was associated with higher obesity rates. Further empirical findings and practical implications are discussed below.

First, the average BMI of boys was higher than that of girls in each age group. This finding was not surprising, as it was consistent with previous studies showing that the average BMI was higher in boys than in girls among Taiwanese adolescents aged 10–18 years from 1997 to 2013^37^. However, it is worth noting that the average BMI of both boys and girls in the 2014 school year was higher than the average BMI reported in the survey data from 1997, 2003, 2008, and 2013. This may suggest that the BMI composition has slightly changed, potentially reflecting a recent increase in BMI among adolescents. Moreover, the combined prevalence of overweight and obesity among adolescents in the 2014 school year was 28.1%. Similarly, the prevalence of overweight and obesity among adolescents in boys was higher than that in girls in this study, and this result has also been observed in previous research^18^. Furthermore, the higher prevalence of overweight and obesity in younger than in older adolescents reflects similar results previously obtained in Taiwan^18^.

Although this study indicated that the rising trend in overweight and obesity was lower in Taiwan than in highly developed countries such as the United States^38^, we cannot afford to ignore the growing crisis of adolescent obesity in relatively newly developed countries such as Taiwan. In particular, as the influence of Western culture has grown worldwide in the past few decades, the Western lifestyle has also spread throughout Taiwan at an accelerated speed, leading adolescents to adopt Western eating habits and increasing their likelihood of becoming obese^23,24^. Because obesity during adolescence can be harmful to the body in a number of ways^10,39–41^, future studies could usefully expand on the present study to track the rates of obesity among Taiwanese adolescents to provide the government data needed to monitor obesity prevalence and enhance investments for the purpose of improving obesity prevention.

Next, we discuss the negative relationship between socioeconomic status and obesity among Taiwanese adolescents below. Accumulating evidence indicates an inverse relationship between socioeconomic status and obesity in developed countries but a positive relationship in developing countries^7,11,42^. As mentioned above, little empirical evidence has been provided to clarify the uncertain relationship between socioeconomic status and adolescent obesity in newly developed countries. Our results revealed a negative association between average family income per household and prevalence of adolescent obesity. Furthermore, given the association between physical fitness and adolescent obesity^20^, we controlled for the effects of physical fitness on the impact of socioeconomic status, and the results remained consistent with our previous understanding. Specifically, the results of the present study suggested that Taiwanese adolescents in low socioeconomic status districts have an increased risk of obesity even after controlling for physical fitness.

Because the socioeconomic status is an aggregate variable derived from average family income per household in each district, we could not completely explain the link between socioeconomic status and obesity in a given person. However, these data help reveal the preliminary trends in the relationship between socioeconomic status and obesity, especially in a newly developed country. Additionally, with the diffusion of Western culture, Taiwanese youth tend to accept the Western lifestyle. The increasing trend in adolescent obesity in Taiwan appears to be similar to that in highly developed countries. Thus, following our study, if obesity is associated with different levels of socioeconomic status in each district, we also provide practical implications for public health officials to design programs for each type of city and county targeting the groups with the greatest need to reduce the prevalence of obesity. For example, school-based and community-based interventions have the potential to prevent adolescent obesity in low- and middle-income contexts^28,43–45^ by creating healthy environments, improving diet and physical activity, providing healthy body size information, etc.

Although we provided evidence that socioeconomic status negatively correlated with adolescent obesity in this study, little is known about the mechanisms driving this association. Further research is needed to replicate our study and to address the mechanisms underlying the relationship between socioeconomic status and adolescent obesity in a newly developed country. Moreover, researchers could further explore whether adolescents raised in a low socioeconomic status setting may experience limited resources, which may be conducive to obesity. As such, we can compensate for knowledge gaps to better understand how socioeconomic inequalities result in more adverse risk factors for obesity among those with a low socioeconomic status in newly developed countries.

The main strength of our study was that it offered new insight into how socioeconomic status leads to weight gain among adolescents in the context of economic and social transition. However, several limitations of this study should be noted. First, the study used a cross-sectional design, which limits our ability to make causal inferences between socioeconomic status and obesity. Further research is therefore needed to follow up on the data provided in this database for exploration of the causal relationships between these factors. Second, the absence of data on districts, gender, age, BMI or physical fitness among adolescents was also a limitation. Similarly, these data are likely biased, with an artificially low proportion of adolescents with low socioeconomic status and educational disadvantages because those adolescents’ data were not completely recorded at school. Third, the Taiwan School Physical Fitness Database lacked family background information at the individual level (such as parents’ education level, occupational status, and family income per household); consequently, we aggregated the individual variables to the city and county level and then combined the average family incomes per household for each district to examine the correlations between socioeconomic status and obesity. Although we lacked family background information at the individual level, after controlling for the effects of gender, age, and physical fitness, the findings revealed the same pattern of relationship between socioeconomic status and obesity. Despite these limitations, the current study not only represents an important starting point for our understanding of the relationship between socioeconomic status and obesity among Taiwanese adolescents but also provides scientific guidance to public health policy makers.

## Conclusions

In conclusion, Taiwanese adolescents currently have a higher risk of overweight and obesity than in the past. In particular, young adolescents are more likely to be obese than older adolescents. Moreover, this study demonstrated a negative relationship between socioeconomic status and obesity among adolescents. This improved understanding of the associations between socioeconomic status and adolescent overweight and obesity will help in the prevention and management of adolescent obesity, thus improving public health. Future work should follow up on the trends in the prevalence of adolescent overweight and obesity and should further explore the mechanisms driving the association between socioeconomic status and obesity across countries at different levels of development.
