# Author Correction: Investigating the relationship between district-level socioeconomic status and individual obesity in Taiwanese adolescents: A large-scale cross-sectional analysis

**DOI:** 10.1038/s41598-020-59136-7

**Published:** 2020-02-06

**Authors:** Ying-Lien Ni, Jen-Ho Chang, Lung Hung Chen

**Affiliations:** 1 Department of Physical Education, Health & Recreation, National Chiayi University, Chiayi, Taiwan; 2 Institute of Ethnology, Academia Sinica, Taipei, Taiwan; 3 Department of Psychology, National Taiwan University, Taipei, Taiwan; 4 Department of Recreation and Leisure Industry Management, National Taiwan Sport University, Taoyuan, Taiwan

Correction to: *Scientific Reports* 10.1038/s41598-019-39167-5, published online 27 February 2019

In Figure 3, the full district names are missing. The correct Figure 3 appears below as Figure 1.

**Figure 1:**
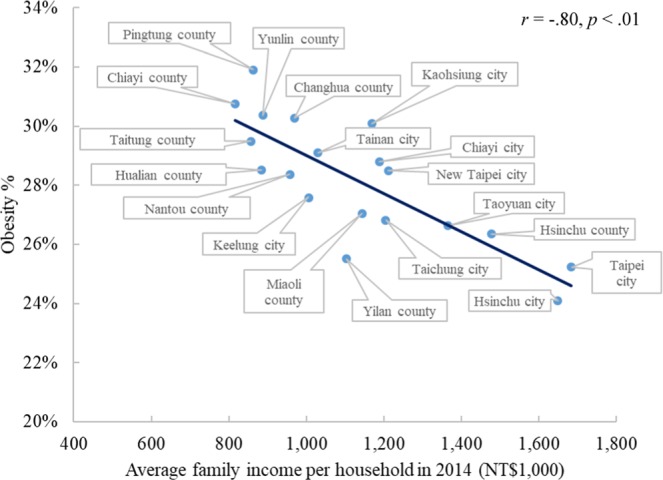
.

